# Cascaded Deep Learning Neural Network for Automated Liver Steatosis Diagnosis Using Ultrasound Images

**DOI:** 10.3390/s21165304

**Published:** 2021-08-05

**Authors:** Se-Yeol Rhyou, Jae-Chern Yoo

**Affiliations:** College of Information and Communication Engineering, Sungkyunkwan University, Suwon 440-746, Korea; fbtpduf@naver.com

**Keywords:** fatty liver, liver steatosis, ultrasound image, semantic segmentation, CNN

## Abstract

Diagnosing liver steatosis is an essential precaution for detecting hepatocirrhosis and liver cancer in the early stages. However, automatic diagnosis of liver steatosis from ultrasound (US) images remains challenging due to poor visual quality from various origins, such as speckle noise and blurring. In this paper, we propose a fully automated liver steatosis prediction model using three deep learning neural networks. As a result, liver steatosis can be automatically detected with high accuracy and precision. First, transfer learning is used for semantically segmenting the liver and kidney (L-K) on parasagittal US images, and then cropping the L-K area from the original US images. The second neural network also involves semantic segmentation by checking the presence of a ring that is typically located around the kidney and cropping of the L-K area from the original US images. These cropped L-K areas are inputted to the final neural network, SteatosisNet, in order to grade the severity of fatty liver disease. The experimental results demonstrate that the proposed model can predict fatty liver disease with the sensitivity of 99.78%, specificity of 100%, PPV of 100%, NPV of 99.83%, and diagnostic accuracy of 99.91%, which is comparable to the common results annotated by medical experts.

## 1. Introduction

Early diagnosis and treatment of liver steatosis, defined as the abnormal accumulation of fat in more than 5% of liver cells, are critically important [1] to prevent further progression of liver diseases, such as hepatocirrhosis and hepatocellular carcinoma [2,3,4]. Ultrasound (US) is the most widely used imaging technique, particularly for diagnosing liver steatosis [5,6]. However, US images are inevitably degraded by speckle noise, blurring, shading, and other artifacts, which cause adverse effects, sometimes leading to misdiagnosis based on image interpretation [7,8]. The US image quality strongly depends on how effectively the speckle noise is reduced. Thus, many attempts have been made to reduce speckle noise and improve visual quality for better diagnoses [9,10,11,12,13,14,15]. However, despite the improved performance, these methods still suffer from several limitations as they are sensitive to the selected kernel or prone to image blurring. In addition to reducing speckle noise, much work has been carried out to assess the level of liver steatosis more precisely by applying complicated algorithms, statistical models, or image-processing techniques to US images. Among these, the hepatorenal index (HRI) and the gray-level co-occurrence matrix (GLCM) are the most commonly known accurate, simple, and cost-effective tools used in the screening for liver steatosis [16,17,18,19]. However, these methods significantly depend on the skill of choosing the region of interest (ROI) and the experience of physicians performing the examination.

Recently, several deep learning-based artificial intelligence approaches have been introduced in the literature [20,21,22,23,24,25] to overcome the issues and challenges associated with US image quality and operator dependency. Andrea et al. [20] proposed a computer-aided diagnosis (CAD) system based on feature extraction to assist in the classification task of liver pathologies. The incorporated feature extraction is based on first-order statistics, co-occurrence matrix, run-length matrix, and fractal dimensions, where three different classifiers are used for the evaluation of certain features, including artificial neural network, support vector machine (SVM), and k-nearest neighbor. However, the CAD system achieved the accuracy of 79.77%, which is not sufficient for automated clinical use. In addition, Zhang et al. [21] used a shallow convolutional neural network (CNN)-based model to extract texture features from US images and detect liver steatosis levels. Their experiment was generally based on the unrealistic assumption that the texture of a normal liver US image is uniform, while that of the fatty liver is nonuniform. The actual liver US images acquired from a commercial scanner are too obscure and shaded to confidently classify fatty liver using such a shallow CNN-based model. A deep CNN pretrained through transfer learning was first applied by Byra et al. [22] and compared with the HRI and GLCM, showing that the pretrained CNN produces a better result. Transfer learning is a machine learning technique where a model trained on one task is re-purposed on a second related task. It makes use of the knowledge gained while solving one problem and applying it to a different but related problem [26,27]. However, considering the performances of the HRI and GLCM, which are greatly affected by the selection of ROIs, it is difficult to believe that the pretrained CNN outperforms the HRI- and GLCM-based classification methods. Cao et al. [23] compared three image-processing techniques: envelope signal, grayscale values, and a neural network. Although the comparison showed that the neural network had the best sensitivity and specificity in assessing the severity of nonalcoholic fatty liver disease, the result of a deep learning neural network was not considered. They used a shallow network architecture with only three convolutional layers and two fully connected layers for the experiment.

As an actual deep learning approach for assessing liver steatosis, a previous study [24] used transfer learning with two pretrained networks, VGG16 and Inception v3, which are currently the most preferred models of deep learning neural networks. According to the results, the transfer deep learning exhibits high accuracy and sensitivity in classifying normal and fatty liver images. Nevertheless, further studies are required for automated patch selection because the assessment still requires the use of patches manually chosen according to physician preference, which could have significantly influenced the results of fatty liver estimates. More recently, research using deep learning networks has been conducted, but some limitations remain. Zamanian et al. [25] used four pretrained networks, specifically Inception v2, GoogLeNet, AlexNet, and ResNet101, to extract features from initial data. All features from the four networks were then summed and classified using an SVM. The results were compared with those from the four individual networks. Although improved results were expected, the actual experimental results show that the individual pretrained networks are more accurate than the proposed algorithm combining the four networks. Specifically, AlexNet and ResNet101 produce better results, but they still contain errors.

In the present work, a cascaded deep learning neural network is proposed to automatically estimate the level of liver steatosis from a US image. The model constitutes three deep learning neural networks for liver and kidney (L-K) detection, ring detection, and grading the severity of disease (i.e., SteatosisNet).

(a) L-K detection involves cropping of the L-K area from a given US image. To achieve this, the DeepLabv3+ model [28] is employed for the semantic segmentation [29] of L-K candidate areas. This is combined with transfer learning to speed up the training and improve the performance of the model.
(b) Ring detection involves checking the L-K area obtained from a US image by checking for the presence of a ring that typically appears around the kidney. This method is employed for areas that are difficult to detect using only the L-K detection method described above.
(c) SteatosisNet takes the above L-K areas as the input and grades the severity of fatty liver disease. It incorporates transfer learning using a CNN model called Inception v3 [30] with a dataset comprising the obtained cropped L-K areas.

We present very promising results regarding the accuracy, sensitivity, and specificity of the proposed model using a dataset from the Samsung Medical Center (SMC) and the widely adopted Byra dataset [22]. The rest of the paper is organized as follows. Data preparation and preprocessing are explained in Section 2.1 and Section 2.2, respectively. In Section 2.3, the proposed cascaded deep learning neural network is briefly described. Section 2.4 and Section 2.5 present the L-K detection and ring detection results, respectively. Using the experimental results, the quality of the proposed cascaded deep learning neural network is illustrated in Section 3. Finally, the work is concluded in Section 4.

## 2. Materials and Methods

### 2.1. Dataset Preparation

**Figure 1:**
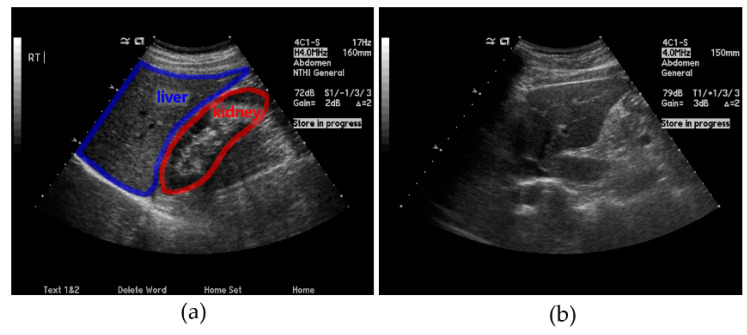
Examples of ultrasound (US) images: (**a**) parasagittal and (**b**) non-parasagittal.

The liver US images used in this study were collected using a Siemens ACUSON Sequoia 512 US machine, with the frequency range of 3–6 MHz, 256 Gy levels, and maximum depth of 36 cm, from the SMC, which is one of South Korea’s leading hospitals. In addition to this main dataset, we collected liver US images from a public dataset [22]. The whole dataset comprised 3200 images obtained in the parasagittal scanning plane. As shown in Figure 1, the parasagittal scanning plane is where most liver parts, the right kidney, and the diaphragm are well-visualized in US imaging.

Medical experts previously annotated the images as normal, mild, moderate, or severe, according to the level of steatosis. Then, the US image dataset was randomly split into training, validation, and test sets with a 6:2:2 ratio, respectively, as listed in Table 1, where the training set was used to build an optimized network model through supervised learning to label unknown test examples.

### 2.2. Preprocessing

**Figure 2:**
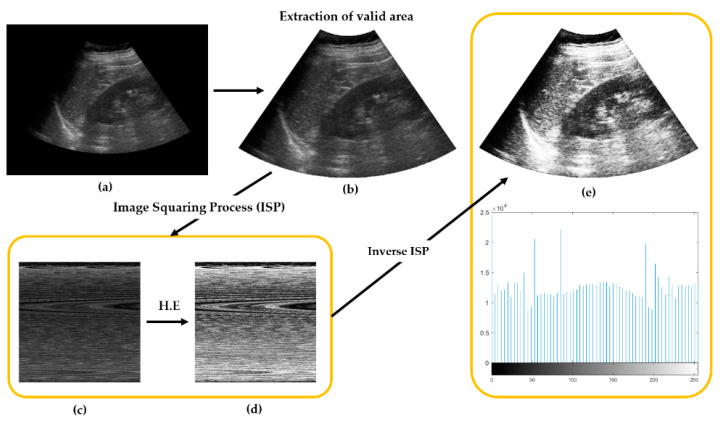
Overview of preprocessing steps: (**a**) Original US image. (**b**) Image with black parts removed. (**c**) Image is geometrically rearranged into a squared image via ISP. (**d**) Histogram equalization (HE) of image in (**c**). (**e**) Reverse conversion of image into the original shape via inverse ISP, and its histogram result.

The images were resized to 960 px × 720 px and converted to the PNG file format for inputting to our deep learning network. Then, all metadata and unnecessary black parts were removed from the dataset before applying histogram equalization (HE), as shown in Figure 2. HE is a useful image-processing technique that adjusts image intensities to enhance contrast between medical devices [31]. This allows our deep learning model to maintain better compatibility with images from different equipment or existing databases.

### 2.3. Proposed Cascaded Deep Learning Neural Network

**Figure 3:**
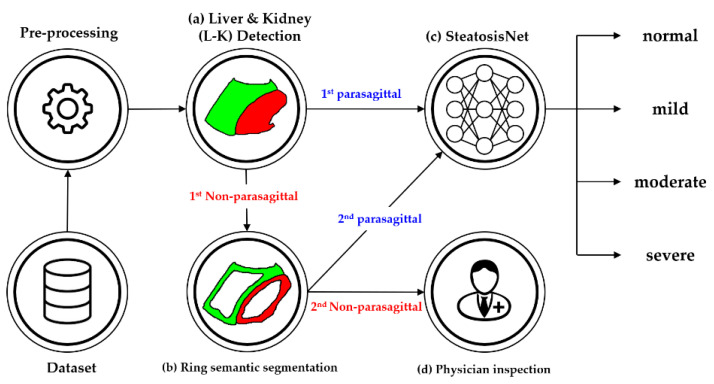
Simplified flowchart of the proposed method: (**a**) Liver and kidney (L-K) detection yielding 1st parasagittal and non-parasagittal images. (**b**) Ring detection to double-check the 1st non-parasagittal image, producing 2nd parasagittal and non-parasagittal images. (**c**) Grading of the 1st and 2nd parasagittal images using SteatosisNet according to the level of steatosis. (**d**) Grading the steatosis level of the 2nd non-parasagittal images by physician inspection.

The proposed cascaded deep learning neural network is shown in Figure 3. It consists of three cascaded neural networks.

(i) L-K detection: In this step, a pretrained deep learning neural network was used for cropping the L-K area while classifying parasagittal and non-parasagittal images.
(ii) Ring detection: This step checks the parasagittal images via so-called “ring semantic segmentation (RSS),” where the presence of a ring, that is typically located around the kidney, was determined in the images.
(iii) Liver steatosis grading: The SteatosisNet used an Inception V3 network [32] transfer-learned with cropped L-K images. Once being transfer-learned, the liver and kidney areas obtained from the above steps (i) and (ii) were taken as the input, and the grade of liver steatosis was determined.

The following sections detail the steps required for L-K detection, ring detection, and liver steatosis grading.

### 2.4. Liver and Kidney (L-K) Detection

**Figure 4:**
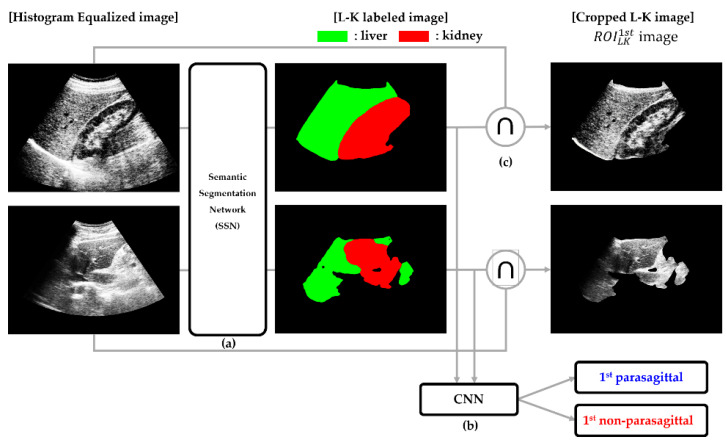
Flowchart of L-K detection: (**a**) Semantic segmentation for cropping of L-K area; (**b**) CNN for the classification of 1st parasagittal and non-parasagittal images; (**c**) Masking operation to yield a cropped L-K image (${\mathrm{ROI}}_{\mathrm{LK}}^{1\mathrm{st}}$), where the ${\mathrm{ROI}}_{\mathrm{LK}}^{1\mathrm{st}}$ image indicates the cropped US image considered as the L-K area from the semantic segmentation result.

In this step, a semantic segmentation network (SSN) was used to localize and crop the L-K area, while a CNN was employed to classify the US images into two categories: parasagittal and non-parasagittal. Accordingly, a novel L-K detection method was designed, such that it cascades the SSN to the CNN. Figure 4 illustrates cropping of the L-K area and determination of images as parasagittal or not through the serial connection of the SSN and CNN. The steps involved in L-K detection are summarized as follows.

(a) Cropping of the L-K area: SSN was employed to obtain an L-K labeled image from a given HE image.
(b) Classifying 1st parasagittal and non-parasagittal images: The output of the SSN was used as the input for the CNN, which then classified the L-K labeled image as a parasagittal or non-parasagittal image.
(c) Masking operation: The logical AND operation between the L-K labeled area and HE image yielded the cropped L-K image (${\mathrm{ROI}}_{\mathrm{LK}}^{1\mathrm{st}}$).

#### 2.4.1. Cropping L-K Area

**Figure 5:**
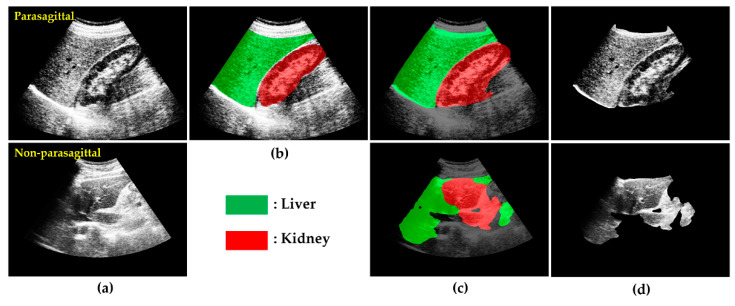
Some results of L-K semantic segmentation and L-K image cropping: (**a**) HE images. (**b**) Ground truth of US image with L-K area labeled. (**c**) L-K labeled images overlaid onto those in (**a**), with liver highlighted in green and kidney in red. (**d**) ${\mathrm{ROI}}_{\mathrm{LK}}^{1\mathrm{st}}$ images. Top row: parasagittal; Bottom row: non-parasagittal.

Localizing and cropping the L-K cortex on a US image was the most important step in our study because it offered crucial and rich information for predicting the level of liver steatosis. For this, we used a DeepLabv3+ network pretrained through transfer learning that helped to segment the L-K area more effectively where the pretrained network was further trained on the specific target of interest, such as the liver and kidney. First, the DeepLabv3+ network was initialized with the weights from a pretrained ResNet-18 network and then transferred to the L-K labeled dataset (total of 2.650 images) to obtain a new classifier for segmenting the L-K area. Figure 5 presents some semantic segmentation results when the transfer learning network was applied for both parasagittal (top row) and non-parasagittal (bottom row) images. Figure 5b shows the ground truth for the two classes, liver and kidney, labeled with different colors. Meanwhile, Figure 5c shows the corresponding L-K labeled images overlaid onto the original HE images. When the L-K labeled images were obtained, the L-K area, herein referred to as the “ROI_LK_ image,” could be logically cropped via the masking operation, defined as

ROI_LK_ image = HE image = HE image ∩ L-K labeled area, (1)

where ∩ indicates the operator that performs the masking operation between the HE images and L-K labeled area. In this figure, it is also apparent that the image on the bottom row, compared with those on the top row, is much less segmented into red or green because it is a non-parasagittal image. Thus, the ROI_LK_ images are easily classified into parasagittal and non-parasagittal images using the CNN, which is trained by supervised learning with a training set collected from the L-K labeled images manually annotated as parasagittal or non-parasagittal.

#### 2.4.2. Classifying Parasagittal and Non-Parasagittal Images

**Figure 6:**
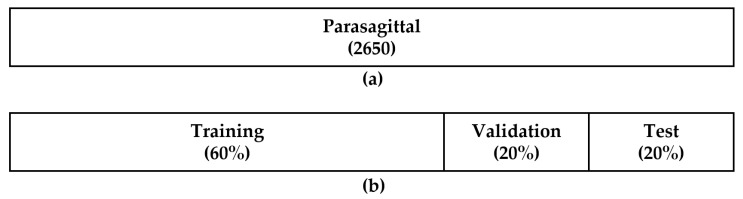
Datasets for the transfer learning of Inception v3: (**a**) Dataset consists of 2650 parasagittal images. The dataset was obtained by collecting the L-K labeled images from the SMC dataset, as listed in Table 1, and then divided into (**b**) training (60%), validation (20%), and testing (20%) sets regarding steatosis level.

When the L-K labeled images were obtained, they were inputted to the CNN for classification as parasagittal and non-parasagittal images, resulting in the first parasagittal and non-parasagittal images. If an L-K labeled image was classified as a parasagittal image with high possibility, then the corresponding HE image was categorized as a parasagittal image. For this purpose, a CNN model called Inception v3 was transfer learned using a dataset of L-K labeled images. Figure 6 shows the data split between training, validation, and test sets for the transfer learning of Inception v3, where each set consists of parasagittal and non-parasagittal L-K labeled images. The resulting transfer-learned network achieved the accuracy of 99.90% for parasagittal detection on the test set.

#### 2.4.3. Masking Operation

**Figure 7:**
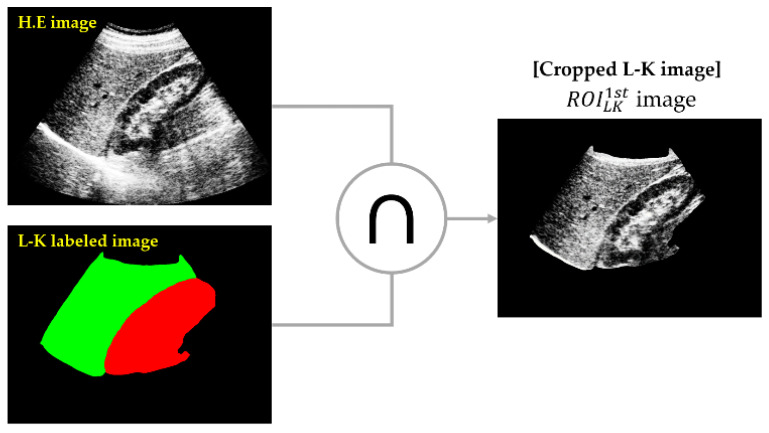
Masking operation (∩) on L-K labeled area and HE image to yield the cropped L-K image.

As shown in Figure 7, the masking operation was used to extract the L-K regions from the HE images by taking the logical AND operation on the L-K labeled area and HE image, yielding the cropped L-K image (${\mathrm{ROI}}_{\mathrm{LK}}^{1\mathrm{st}}$). The masking operation removes all unnecessary components in assessing the steatosis level of the liver, except the L-K regions, and thus provides better prediction of liver steatosis severity.

The ${\mathrm{ROI}}_{\mathrm{LK}}^{1\mathrm{st}}$ images were inputted to SteatosisNet for grading the severity of fatty liver disease as normal, mild, moderate, or severe. As will be explained in Section 3.2, compared with when non-cropped images were applied, the use of cropped L-K images improved the grading accuracy by approximately 4.5%. This was mainly because SteatosisNet can pay more attention to L-K features cropped by semantic segmentation [32], without irrelevant information.

### 2.5. Ring Detection

**Figure 8:**
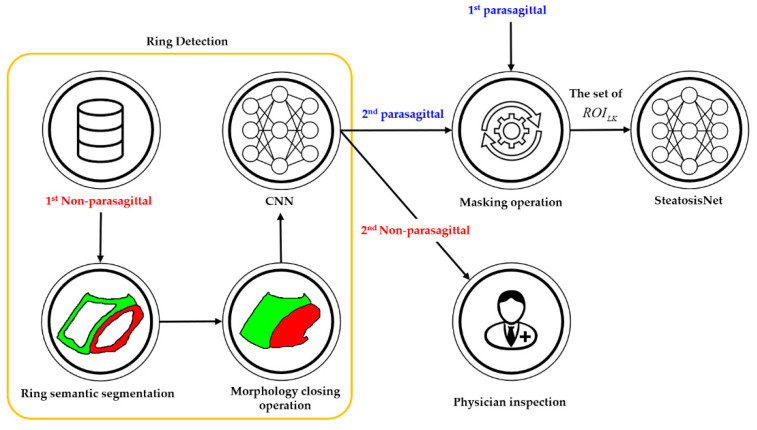
Simplified flowchart of ring detection, where physicians can inspect the 2nd non-parasagittal images to grade the steatosis level. Ring segmentation produced a ring-segmented image, and the morphology closing operation yielded an L-K labeled image. The set of ROI_LK_ images was inputted to SteatosisNet.

Ring detection was a further step for identifying parasagittal images that might have been missed during L-K detection. Therefore, the input of ring detection would represent the 1st non-parasagittal image. One of the outstanding features of parasagittal images is the ring-shaped contour encircling the kidney cortex; thus, if a ring-shaped contour can be found on a US image, then it is most likely a parasagittal image. As described in Figure 8, ring detection includes two steps. The first step includes RSS, which is a type of semantic segmentation for identifying ring objects at the pixel level on a given 1st non-parasagittal image. To achieve this, DeepLabv3+ was transfer learned using the same parasagittal training set presented in Figure 6, but labeled with two ring objects, each encircling the liver or kidney cortex.

**Figure 9:**
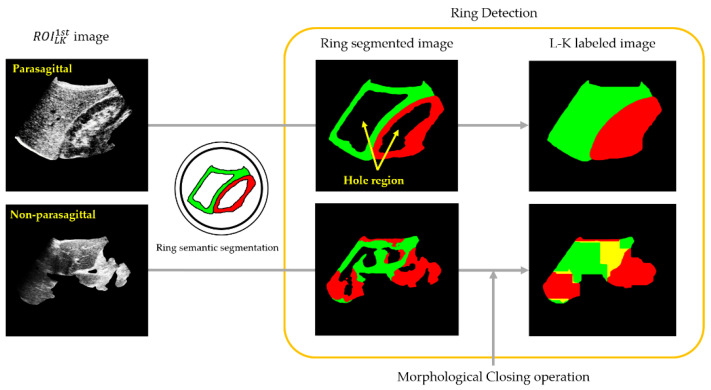
Example of ring semantic segmentation and morphological closing operation for a given ${\mathrm{ROI}}_{\mathrm{LK}}^{1\mathrm{st}}$ image.

After the two rings were semantically segmented on the US image, their inner portions (i.e., hole regions) could be completely filled with the corresponding color labels to readily produce an L-K labeled image. Therefore, the hole-filling process of the ring-segmented image is the second step in which the morphological closing operation [33] was applied to the ring-segmented image, resulting in an L-K labeled image. As shown in Figure 9, an L-K-labeled image derived from a parasagittal image was more likely to be parasagittal. Finally, the CNN and masking operation, as described in Section 3.2 and Section 3.3, could again be applied to the L-K labeled image to obtain the 2nd parasagittal and the corresponding ${\mathrm{ROI}}_{\mathrm{LK}}^{2\mathrm{nd}}$ images, forming the set of ${\mathrm{ROI}}_{\mathrm{LK}}$, given by

(2) $${\mathrm{ROI}}_{\mathrm{LK}}=\left\{{\mathrm{ROI}}_{\mathrm{LK}}^{1\mathrm{st}},{\;\mathrm{ROI}}_{\mathrm{LK}}^{2\mathrm{nd}}|\mathrm{US}\;\mathrm{Image}\;\in \;\mathrm{parasagittal}\right\},\;$$

**Figure 10:**
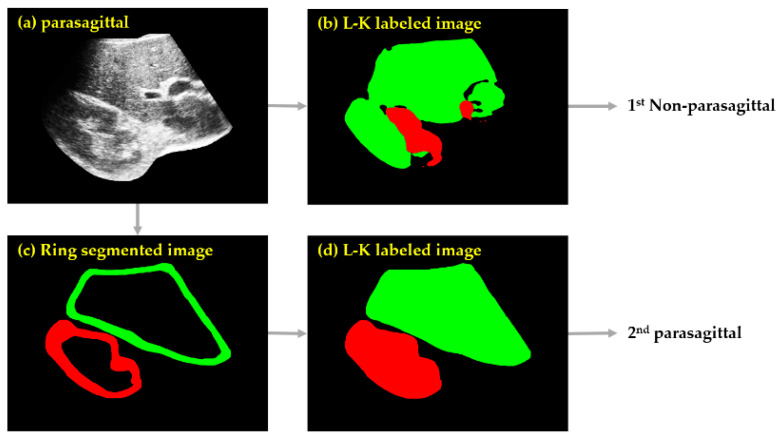
Example of 2nd parasagittal image with ring detection: (**a**) HE US image. (**b**) L-K labeled image, misclassified as a 1st non-parasagittal image. (**c**) Ring-segmented image obtained via ring detection. (**d**) L-K labeled image, which was parasagittal, with the application of morphology closing operation to the result in (**c**).

Figure 10 shows an example of the effectiveness of ring detection, where a 1st non-parasagittal image, which should be parasagittal, could be reclassified as parasagittal through ring detection. According to the experimental results, the detection accuracy of parasagittal images increased by 0.07% upon the application of ring detection, and hence a very high performance was achieved. The set of ${\mathrm{ROI}}_{\mathrm{LK}}$ images was inputted to SteatosisNet for grading the severity of fatty liver disease as normal, mild, moderate, or severe.

## 3. Results

The proposed deep learning model was implemented with MATLAB programming language on a machine with a 2-way GeForce RTX 2080 Ti GPU 11GB. Liver US images were collected from the SMC, and a public dataset (https://zenodo.org/record/1009146#.YL2a5fkzYuU (accessed on 21 May 2021), [24]) to verify the performance of the proposed cascaded deep learning model. The images were categorized based on the level of disease severity: normal, mild, moderate, or severe. In addition, data augmentation techniques [34] were used to generate more training data, where affine transformations, such as a random rotation of ±20 and random translation of ±5 pixels in the horizontal/vertical direction, were applied to the original dataset. These data augmentations help avoid overfitting issues while training. As shown by the experiments, the proposed cascaded deep learning neural network yields better performance than the recently reported results (see Table 2), confirming the advantages of the proposed model. The results of L-K detection, RSS, and SteatosisNet are described in detail below.

### 3.1. Performances of L-K Detection and Ring Semantic Segmentation

The resulting performances of semantic segmentation related to L-K detection and ring detection are presented in Table 2. In this work, a cross-entropy loss was used when adjusting the model weights during training of the neural networks, while the semantic segmentation quality was evaluated using metrics, such as the mean accuracy, mean intersection over union (IOU), and boundary F-1 score (BF1 score), as shown in Table 2.

**Table 2:** Semantic segmentation performances of L-K detection and ring detection (IOU: intersection over union, BF1: boundary F-1).

Performance Deleted Extra Space					
	Area	Mean Accuracy	Mean IOU	BF1 Score	Dataset
L-K Detection	Kidney	0.9682	0.8088	0.4650	Training: 1590 Validation: 530 Test: 530 N:Mi:Mo:S = 30:11:10:2
	Liver	0.9487	0.7856	0.5228	
	Null	0.9415	0.9341	0.8002	
Ring Detection	Kidney	0.8642	0.6665	0.5510	
	Liver	0.8318	0.6576	0.5785	
	Null	0.8318	0.8307	0.8663	
Training optimizer: Adam, Minibatch size: 8, Max epoch: 10, Learning rate of 0.001 with decay factor of 0.9, Termination condition: validation accuracy < 0.98					

The mean IOU is a common evaluation metric for image semantic segmentation and quantifies the percentage overlap between the ground truth and predicted pixels, whereas the BF1 score is a measured value of how close the boundaries of segmented images match those in the ground truth. Table 2 shows that the BF1 score was relatively low compared with the mean accuracy or the mean IOU. This is because speckle noise is inherently present in medical US images. Fortunately, in assessing liver steatosis, echogenicity and echotexture from the L-K areas are much more important than the boundary. The BF1 score merely indicates how well the predicted boundary aligns with the true boundary, and hence does not significantly affect the prediction accuracy of hepatic steatosis. Therefore, it makes sense to improve the IOU or accuracy metrics rather than the BF1 score by either adjusting training parameters or augmenting data. The mean accuracy, IOU, and BF1 score were lower for ring detection than for the semantic segmentation of the L-K area, but this is not very important because ring detection only determines the edge of the liver and kidney. It was also found that the overall detection accuracy of parasagittal images could be improved by up to 99.97% upon ring detection.

### 3.2. Performance of SteatosisNet

**Figure 11:**
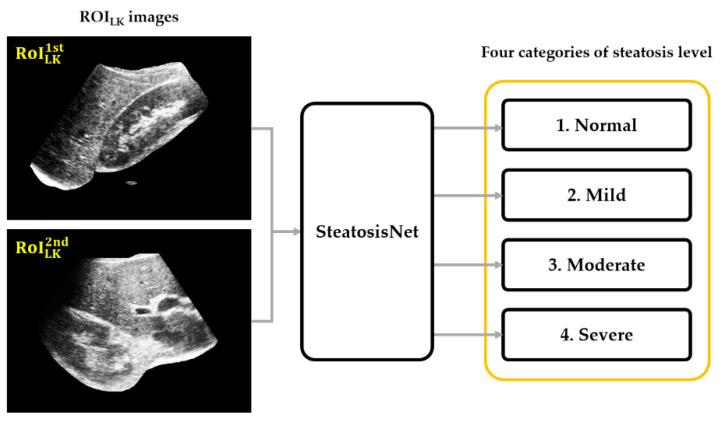
Flowchart of SteatosisNet classification.

As shown in Figure 11, SteatosisNet classifies ${\mathrm{ROI}}_{\mathrm{LK}}$ images, each falling into one of four categories regarding the level of steatosis: normal, mild, moderate, or severe.

**Figure 12:**
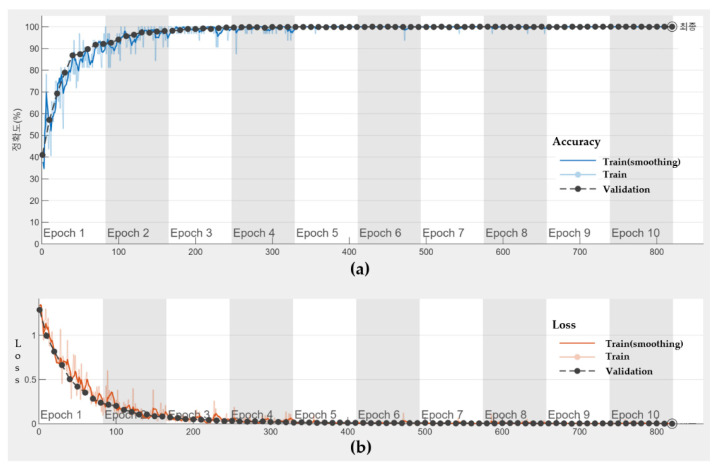
Performance of SteatosisNet on training and validation sets of SMC dataset: (**a**) Accuracy; (**b**) Loss.

SteatosisNet uses the CNN model Inception v3, transfer-learned with 2,0 ROI_LK_ images, to grade the severity of fatty liver disease. The ROI_LK_ images were split into four categories according to the steatosis level: normal (0–5%), mild (5–30%), moderate (30–70%), and severe (70–100%). The model was trained with the Adam (adaptive moment estimation) optimizer, along with momentum (momentum rate = 0.9). The initial learning rate was set as 0.001. The max epoch with the termination condition of validation accuracy of <99.98 was set as 10 to guarantee sufficient training of the model as well as mitigate network overfitting. Early stopping is a technique used to terminate the training before overfitting occurs. The training terminates immediately when the termination condition is satisfied. Shuffling of the training data is applied at the beginning of every epoch to help the model converge on the optimal solution sooner. Figure 12a shows the training and validation accuracy (*y*-axis) over 10 training epochs (*x*-axis). The corresponding loss is displayed in Figure 12b. Note that the validation set is not used to update the network weights, but to assess whether a model suffers from overfitting. As shown in Figure 12b, the training progress stops early at epoch 10. This is when the validation accuracy is below 99.98%.

**Figure 13:**
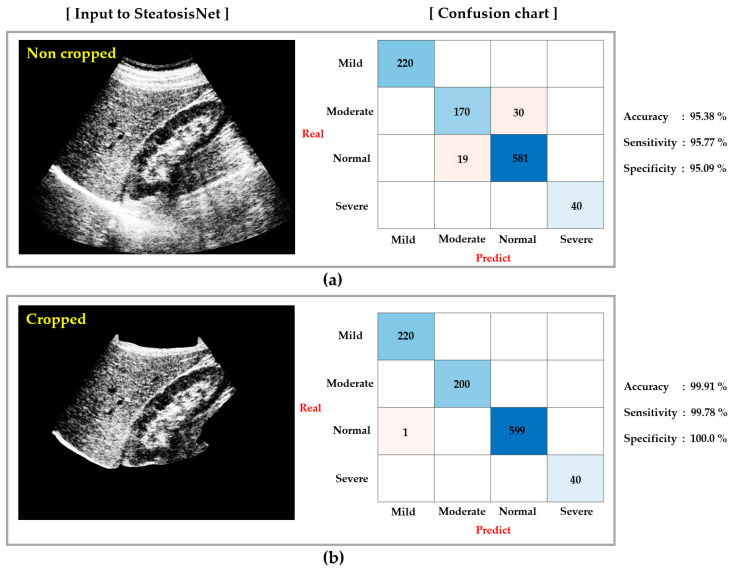
Performance comparison between use of (**a**) non-cropped and (**b**) cropped images for the SMC dataset.

The experimental results were assessed using performance evaluation metrics, including the classification accuracy, sensitivity, and specificity. The analytical comparison in Figure 13 shows how much the performance of SteatosisNet is improved with use of the cropped L-K image compared with the non-cropped image. The performance evaluation metrics improved by approximately 4–5% on average when cropped US images were used as the input to SteatosisNet. This is because the cropped L-K image does not contain unnecessary parts; therefore, SteatosisNet can focus more on liver steatosis-related areas, leading to better results.

The proposed model was compared with state-of-the-art results provided in the published literature [22,23,24,25,26,27]. As seen in Table 3, the proposed model is the best regarding various performance evaluation metrics, such as accuracy, sensitivity, and specificity. The resulting metrics based on the testing dataset reached almost 99–100%. It is also apparent from Table 3 that the proposed model exhibits almost the same performance for both the SMC and Byra datasets. Thus, it can be concluded that the proposed cascaded neural network model is fairly robust between databases and between different US image qualities. The results of this study reveal that the proposed model can serve as a valid and reliable screening tool for estimating the level of steatosis, and for identifying patients who require further investigation.

### 3.3. Ablation Study of Our Method on SMC Database

We designed an ablation study to examine the power of ring and L-K detections in an SMC dataset of US images. SteatosisNet uses a deep convolutional neural network deciding the steatosis level at the final stage, thus it is definitely essential in our study. In this study, it compares the performance of our network with the following configurations:

(1) Ring detection is ablated, i.e., only L-K detection is taken into account; (2) L-K detection is ablated, thus only ring detection; and (3) both ring and L-K detections are ablated, thus only SteatosisNet is considered. These ablated architectures are trained under the same training scheme and tested with the same data.

The following table shows the ablation study results on the dataset.

The accuracy metric has been widely used for evaluating the classification models. The metric calculates the proportion of correctly classified instances, either true positives or true negatives. Equation (3) represents the accuracy where TP stands for true positives, TN for true negatives, FP for false positives and FN for false negatives.

(3) $$\mathrm{Accuracy}\;=\frac{\mathrm{TP}\;+\mathrm{TN}}{\mathrm{TP}\;+\;\mathrm{FP}\;+\mathrm{TN}\;+\;\mathrm{FN}}$$

From the Equation (3), it is seen that the simplest way to improve the accuracy is to decrease FP and FN. Remember that when we crop and extract only liver and kidney areas through L-K detection, which are the most informative portion of US images in estimating the steatosis level, we could decrease FP and FN by 97.96% (See Figure 13), eventually leading to an improvement in accuracy. Table 4 quantitatively proves how much the cropping technology can help improve the overall performance where the test performance largely degrades as the ablation happens on the L-K detection. Therefore, we find that the L-K detection is highly necessary in our system. Additionally, the above table indicates that, on average, the ring detection is substantially effective in reducing the screening inspection cost. The above ablation study results teach us that the L-K detection is relatively more significant than ring detection as a new component in improving performance, nevertheless both are useful in implementing an effective liver steatosis diagnosis system.

## 4. Discussion and Conclusions

US images are the most commonly used type of image in CAD systems for diagnosing fatty liver disease. In this paper, we proposed a cascaded deep learning neural network model that can automatically predict the level of liver steatosis. The validity of the proposed model was thoroughly evaluated using both the Samsung Medical Center dataset and the Byra database, which is widely adopted in extant studies. Using an effective semantic segmentation of the liver and kidney, the automatic diagnosis task could be effectively accomplished via the masking operation and ring detection. Furthermore, the cascaded deep learning network model exhibited excellent performance in terms of sensitivity, specificity, and accuracy in predicting the level of liver steatosis. We achieved an accuracy of 99.91%, sensitivity of 99.78%, and specificity of 100%, which are incomparable to those of the conventional research results, clearly highlighting its usefulness and feasibility as a screening tool for grading liver steatosis. We believe that this surprising result is due to the incorporation of the masking operation and ring detection. The former method removes all unnecessary components, except the L-K regions, before assessing the steatosis level, while the latter minimizes the screening number of US images to be inspected by a physician.

The masking operation, which takes only the L-K areas and applies to the input of SteatosisNet, gives a remarkably good result compared with the annotation consistency by medical experts and thus outperforms the state-of-the-art techniques. The masking operation elaborately eliminates non-liver and kidney portions in evaluating and monitoring levels of hepatic steatosis, thus being able to obtain a better prediction of the severity of the fatty liver disease. The ring detection, which tries to detect a ring-shaped contour on US images, increases the detection accuracy of parasagittal images by 0.07% and can accordingly reduce the screening inspection cost. Screening an entire US image is labor-intensive and time-consuming for physicians. The proposed model does not require a presence of physician; in turn, they can invest time into more important tasks and manage patients in critical condition. Thus, the proposed model is promising and can be widely applicable for screening inspection of fatty liver on US images, with a performance comparable to that of physicians.

However, our method has a limitation. The algorithm only works on ultrasound images which are captured by the same ultrasonography machine. It means the network transfer learned with SMC datasets only work at SMC datasets. However, this does not mean that we cannot use other types of ultrasonography images. We need an extra training process when we change the ultrasonography machine. In the future, when different kinds of data are stacked and learned, it will work on images taken from all kinds of ultrasound devices. We expect our new method to be used or helped clinically by radiologists.

## Figures and Tables

**Table 1:** Data source and the number of individual images according to the level of steatosis (N: Normal, Mi: Mild, Mo: Moderate, S: Severe).

Data Source	Training (60%)				Validation (20%)				Test (20%)				US Machine
	N	Mi	Mo	S	N	Mi	Mo	S	N	Mi	Mo	S	
Sasung	900	330	300	60	300	110	100	20	300	110	100	20	ACUSON Sequoia 512
Byra [22]	102	114	54	60	34	38	18	20	34	38	18	20	GE Vivid E9
Total	1902	444	594	180	634	148	198	60	634	148	198	60	--

**Table 3:** Performance comparison regarding classification accuracy, sensitivity, and specificity (%) with recently published state-of-the-art algorithms.

Reference	Model		Accuracy	Sensitivity	Specificity
Andrea et al. [20]	KNN ^(1)^		74.05%	-	-
Zhang et al. [21]	CNN ^(2)^		90.00%	81.00%	92.00%
Byra et al. [22]	CNN ^(3)^		96.30%	100.00%	88.20%
Cao et al. [23]	CNN ^(4)^		73.97%	-	-
Anca et al. [24]	CNN ^(5)^		93.23%	88.90%	-
Zamanian et al. [25]	CNN ^(6)^		98.64%	97.20%	100.00%
Proposed methods	Cascaded NN	♠	99.91%	99.78%	100.00%
		♥	100.00%	100.00%	100.00%
		♣	99.62%	99.13%	100.00%
		♦	100.00%	100.00%	100.00%

♠: When being trained and tested by SMC database. ♥: When being trained and tested by Byra database. ♣: When being trained both by SMC and Byra database, but tested by SMC database. ♦: When being trained both by SMC and Byra database, but tested by Byra database. (1) (2012) ANN where k-nearest neighbor is better than SVM. (2) (2019) Shallow convolutional neural network-based model to extract texture feature. (3) (2018) Pretrained CNN through transfer learning. (4) (2019) 3 image-processing techniques: including envelope signal, grayscale values and a NN. (5) (2020) Transfer learning with comparison of 2 pretrained networks: VGG16 and inception V3. (6) (2021) Performance comparison study of 4 pretrained networks: Inception v2, GoogleNet, etc.

**Table 4:** Ablation study detailed results on SMC dataset.

Ablated Components	Accuracy	Sensitivity	Specificity
Only Ring	98.50%	98.50%	97.17%
Only L-K	96.89%	97.83%	95.65%
Ring + L-K	95.38%	95.77%	95.09%
Nothing	99.91%	99.78%	100.00%

## Data Availability

Not applicable.

## References

[1] Xiang L., Jia L.S., Shuo H.W., Jing W.Z., Qan Q.C. (2017). Learning to Diagnose Cirrhosis with Liver Capsule Guided Ultrasound Image Classification. Sensors.

[2] Kutlu H., Avcı E. (2019). A Novel Method for Classifying Liver and Brain Tumors Using Convolutional Neural Networks, Discrete Wavelet Transform and Long Short-Term Memory Networks. Sensors.

[3] Farrell G.C., Larter C.Z. (2006). Non-alcoholic fatty liver disease: From steatosis to cirrhosis. Hepatology.

[4] Qian Y., Fan J.G. (2005). Obesity, fatty liver and liver cancer. Hepatobiliary Pancreat. Dis. Int..

[5] Matthew M.Y., Elizabeth M.B. (2014). Pathological aspects of fatty liver disease. Gastroenterology.

[6] Lee D.H. (2017). Imaging evaluation of non-alcoholic fatty liver disease: Focused on quantification. Clin. Mol. Hepatol..

[7] Sudha S., Suresh G.R., Sukanesh R. (2009). Speckle Noise Reduction in Ultrasound Images by Wavelet Thresholding based on Weighted Variance. Int. J. Comput. Theory Eng..

[8] Jian Y., Jingfan F., Danni A., Xuehu W., Yongchang Z., Songyuan T., Yongtian W. (2016). Local statistics and non-local mean filter for speckle noise reduction in medical ultrasound image. Neurocomputing.

[9] FaDa G., Phuc T., ShuaiPing G., LiNa Z. (2014). Anisotropic diffusion filtering for ultra-sound speckle reduction. Sci. China Technol. Sci..

[10] Simone B., Carlo G., Oriol P., Jesepa M., Petia R. (2010). SRBF: Speckle reducing bilateral filtering. Ultrasound Med. Biol..

[11] Charles A.D., LoÏc D., Florence T. (2009). Iterative weighted maximum likelihood denoising with probabilistic patch based weights. IEEE Trans. Image Process.

[12] Pierrick C., Pierre H., Charles K., Christian B. (2009). Nonlocal means-based speckle filtering for ultrasound images. IEEE Trans. Image Process.

[13] Sara P., Mariana P., Cesario V.A., Luisa V. (2012). A nonlocal SAR image de-noising algorithm based on LLMMSE wavelet shrinkage. IEEE Trans. Geosci. Remote Sens..

[14] Nedumaran D., Sivakumar R., Sekar V., Gayathri K.M. (2012). Speckle noise reduction in ultrasound biomedical B-scan images using discrete topological derivative. Ultrasound Med. Biol..

[15] Shan G., Boyu Z., Cihui Y., Lei Y. (2018). Speckle noise reduction in medical ultrasound image using monogenic wavelet and Laplace mixture distribution. Digit. Signal Process..

[16] Richard H.M., Marna E., Edward I.B., Paul M.G. (2012). Hepatorenal Index as an Accurate, Simple, and Effective Tool in Screening for Steatosis. Am. J. Roentgenol..

[17] Webb M., Yeshua H., Zelber-Sagi S., Santo E., Brazowski E., Halpern Z., Oren R. (2009). Diagnostic value of a computerized hepatorenal index for sonographic quantification of liver steatosis. Am. J. Roentgenol..

[18] Robert M.H., Shanmugam K., Dinstein I.H. (1973). Textural features for image classification. IEEE Trans. Syst. Man Cybern..

[19] Andrade A., Silva J.S., Santos J., Belo-Soares P. (2012). Classifier approaches for liver steatosis using ultrasound images. Procedia Technol..

[20] Rivas E.C., Moreno F., Benitez A., Morocho V., Vanegas P., Medina R. (2015). Hepatic Steatosis detection using the co-occurrence matrix in tomography and ultrasound images. Signal. Process Images Comput. Vis..

[21] Lei Z., Haijiang Z., Tengfei Y. (2019). Deep Neural Networks for fatty liver ultrasound images classification. Chin. Control. Decis. Conf..

[22] Michał B., Grzegorz S., Cezary S., Piotr K., Łukasz M., Rafał P., Bogna Z.W., Krzysztof Z., Piotr S., Andrzej N. (2018). Transfer learning with deep convolutional neural network for liver steatosis assessment in ultrasound images. Int. J. Comput. Assist. Radiol. Surg..

[23] Fuzhen Z., Zhiyuan Q., Keyu D., Dongbo X., Yongchun Z., Hengshu Z., Hui X., Qing H. (2020). A Comprehensive Survey on Transfer Learning. arXiv.

[24] Chuanqi T., Fuchun S., Tao K., Wenchang Z., Chao Y., Chunfang L. (2018). A Survey on Deep Transfer Learning. arXiv.

[25] Wen C., Xing A., Longfei C., Chaoyang L., Qian Z., Ruijun G. (2019). Application of Deep Learning in Quantitative Analysis of 2-Dimensional Ultrasound Imaging of Nonalcoholic Fatty Liver Disease. J. Ultrasound Med..

[26] Elena C.C., Anca-Loredana U., Ștefan C.U., Andreea V.I., Lucian G.G., Gabriel G., Larisa S., Adrian S. (2020). Transfer learning with pre-trained deep convolutional neural networks for the automatic assessment of liver steatosis in ultrasound images. Med. Ultrason..

[27] Zamanian H., Mostaar A., Azadeh P., Ahmadi M. (2021). Implementation of Combinational Deep Learning Algorithm for Non-alcoholic Fatty Liver Classification in Ultrasound Images. J. Biomed. Phys. Eng..

[28] Liang-Chieh C., Yukun Z., George P., Florian S., Hartwig A. (2018). Encoder-Decoder with Atrous Separable Convolution for Semantic Image Segmentation. arXiv.

[29] Imad M., Doukhi O., Lee D.-J. (2021). Transfer Learning Based Semantic Segmentation for 3D Object Detection from Point Cloud. Sensors.

[30] Christian S., Vincent V., Sergey I., Jon S., Zbigniew W. (2015). Rethinking the Inception Architecture for Computer Vision. arXiv.

[31] Nema S., Hebatullah M., Asmaa S. (2019). Medical image enhancement based on histogram algorithms. Procedia Comput. Sci..

[32] Nasser A., Amr A., AbdAllah A.E., Ahmed A. (2021). Efficient 3D Deep Learning Model for Medical Image Semantic Segmentation. Alex. Eng. J..

[33] Ravi S., Am K. (2013). Morphological Operations for Image Processing: Understanding and its Applications. Natl. Conf. VLSI Signal. Process. Commun..

[34] Vargas Rivero J.R., Gerbich T., Buschardt B., Chen J. (2021). Data Augmentation of Automotive LIDAR Point Clouds under Adverse Weather Situations. Sensors.

